# Statistical Evidence Suggests that Inattention Drives Hyperactivity/Impulsivity in Attention Deficit-Hyperactivity Disorder

**DOI:** 10.1371/journal.pone.0165120

**Published:** 2016-10-21

**Authors:** Elena Sokolova, Perry Groot, Tom Claassen, Kimm J. van Hulzen, Jeffrey C. Glennon, Barbara Franke, Tom Heskes, Jan Buitelaar

**Affiliations:** 1 Institute for Computing and Information Sciences, Radboud University, Nijmegen, The Netherlands; 2 Department of Human Genetics, Donders Institute for Brain, Cognition and Behaviour, Radboud University Medical Center, Nijmegen, The Netherlands; 3 Department of Psychiatry, Donders Institute for Brain, Cognition and Behaviour, Radboud University Medical Center, Nijmegen, The Netherlands; 4 Department of Cognitive Neuroscience, Donders Institute for Brain, Cognition and Behaviour, Radboud University Medical Center, Nijmegen, The Netherlands; 5 Karakter Child and Adolescent Psychiatry University Centre, Nijmegen, The Netherlands; Philipps-Universitat Marburg, GERMANY

## Abstract

**Background:**

Numerous factor analytic studies consistently support a distinction between two symptom domains of attention-deficit/hyperactivity disorder (ADHD), inattention and hyperactivity/impulsivity. Both dimensions show high internal consistency and moderate to strong correlations with each other. However, it is not clear what drives this strong correlation. The aim of this paper is to address this issue.

**Method:**

We applied a sophisticated approach for causal discovery on three independent data sets of scores of the two ADHD dimensions in NeuroIMAGE (total N = 675), ADHD-200 (N = 245), and IMpACT (N = 164), assessed by different raters and instruments, and further used information on gender or a genetic risk haplotype.

**Results:**

In all data sets we found strong statistical evidence for the same pattern: the clear dependence between hyperactivity/impulsivity symptom level and an established genetic factor (either gender or risk haplotype) vanishes when one conditions upon inattention symptom level. Under reasonable assumptions, e.g., that phenotypes do not cause genotypes, a causal model that is consistent with this pattern contains a causal path from inattention to hyperactivity/impulsivity.

**Conclusions:**

The robust dependency cancellation observed in three different data sets suggests that inattention is a driving factor for hyperactivity/impulsivity. This causal hypothesis can be further validated in intervention studies. Our model suggests that interventions that affect inattention will also have an effect on the level of hyperactivity/impulsivity. On the other hand, interventions that affect hyperactivity/impulsivity would not change the level of inattention. This causal model may explain earlier findings on heritable factors causing ADHD reported in the study of twins with learning difficulties.

## Introduction

### Problem description

Attention-deficit/hyperactivity disorder (ADHD) is a common and highly heritable neurodevelopmental disorder that affects about 5–6% of children worldwide [1, 2]. ADHD persists into adulthood in about 30–50% of the childhood cases, depending on definition of remission [3], and prevalence of ADHD in adults is estimated between 2.5–4.9% [4]. In pediatric populations, ADHD is about 2–3 times more common in boys than girls [5], but gender balance is rather equal in adult populations [6]. The genetics of ADHD is complex [7] and several candidate genes have been associated with ADHD in meta-analyses, among which the dopamine transporter gene *SLC6A3/DAT1 [8]* and dopamine D4 receptor gene *DRD4 [9]*. Genetic variation of the *DAT1* gene may affect the functioning of the dopamine transporter caused by individual variation in regulating levels of dopamine [10, 11]. This alters baseline dopamine tone; which is utilized therapeutically by drugs such as methylphenidate that block the dopamine transporter involved in the recycling of dopamine into neurons. The *DAT1* gene has a differential risk haplotype (formed by a variable number of tandem repeat (VNTR) polymorphisms in the 3’ UTR and in intron 8) associated with childhood ADHD (10R/6R) and adult ADHD (9R/6R) [12, 13]. Similarly, polymorphism in the 7 repeat allele of the DRD4 gene (which is expressed on neuronal dendrites) confers reduced intracellular cAMP signalling following binding of dopamine to dopamine D4 receptors. As such, increased expression of these VNTR polymorphisms in *DAT1* or *DRD4* increases the degree of genetic risk associated with ADHD symptoms. Furthermore, both *DAT1* knockout and *DRD4* knockout transgenic mice demonstrate face validity with documented increases in hyperactivity and impulsivity [14] and reduced behavioral inhibition [15].

As evident from its name, ADHD is characterized by inappropriate and pervasive levels of inattention and/or hyperactivity and impulsivity. Exploratory and confirmatory factor analyses of the core ADHD symptoms defined in the DSM system and assessed by parents and teachers, as well as self-report ratings in adolescents and adults consistently support a distinction between two symptom dimensions: inattention and hyperactivity/impulsivity (see [16] for a review). Inattention and hyperactivity/impulsivity both show high internal consistency and are moderately to strongly correlated (correlation coefficient between .63 and .75), indicating that they constitute separable but substantially correlated dimensions [16]. Inattention is more strongly related to internalizing problems of anxiety and depression and to academic underachievement. In contrast, hyperactivity/impulsivity is linked to peer rejection and externalizing behavioral problems such as oppositional defiant and antisocial behavior [16].

The cause of the strong correlation between the two symptom dimensions of ADHD inattention and hyperactivity/impulsivity is yet unclear. Are these two dimensions two sides of the same coin, i.e., the consequence of a (possibly unknown) common cause, or could it be that one dimension drives the other? This question is relevant to the current literature: some studies assume a bi-factor model to explain the correlation [17], others propose a driving effect of inattention on hyperactivity based on the analysis of twin studies [18].

### Causal discovery from observational data

The standard approach to establish causal relationships is through experimental manipulation or intervention. For example, in order to establish a causal effect of inattention upon hyperactivity/impulsivity, one would need to apply an intervention that only acts upon inattention and then measure its effect on hyperactivity/impulsivity. When analyzing the results of these experiments the Bradford Hill criteria for causation should be taken into account [19]. These criteria specify the conditions necessary to provide evidence of causal relationships. Although in theory such an intervention, e.g., through a well-designed therapy or some novel highly specific medication, could be attainable, we are not aware of any such attempts or studies in the current literature.

That being the case, the emerging field of causal discovery from observational data may provide a powerful alternative [20, 21]. In apparent contradiction with the good old adagio “correlation does not imply causation”, theoretical and experimental studies have shown that, under certain reasonable assumptions, it *is* possible to learn cause-effect relationships from purely observational data. The key insight is that, where a single number such as a mere correlation indeed cannot reveal anything about causal direction, other, more subtle characteristics may contain important directional information. Just considering pairs of variables, these can be found in higher-order moments [22]. In higher-dimensional systems, the seminal work of Turing award winner Judea Pearl [21] and others revealed the close connection between causal relationships and conditional independencies. Since then, causal discovery algorithms have successfully been applied in various domains, and slowly find their way into the biomedical sciences [23–26]. To the best of our knowledge, the current paper is the first to describe an application of causal discovery for the analysis of observational clinical data.

Intuitively, two variables *‘Z’* and *‘Y’* are conditionally independent given *‘X’* if, once the value of variable *‘X’* is known, the value of *‘Z’* does not add any additional information about *‘Y’*. For example, in the context of children with ADHD, we can call gender and hyperactivity/impulsivity conditionally independent given inattention, if knowing whether a subject is a boy or a girl does not help to better estimate the hyperactivity/impulsivity symptom score, once we already know the child’s inattention symptom score. In this paper we investigate whether such conditional independencies can be derived from observational data.

Most causal discovery algorithms start by assuming that real-world events are governed by specific, yet unknown causal mechanisms. Given a particular causal model, one can in principle read off the conditional dependencies and independencies one should then find in observational data. Reasoning backwards, given particular observed conditional dependencies and independencies in observational data, one may be able to infer causal relations that any causal model should have to be consistent with the observed statistical patterns.

It is exactly this kind of inverse reasoning that underlies so-called constraint-based algorithms for causal discovery such as PC/Fast Causal Inference [27] and Bayesian Constraint-based Causal Discovery [28]. Specialized variants, such as Cooper’s local causal discovery algorithm (LCD) [29] and the Trigger algorithm [24], handle the case of three variables and are particularly relevant for our purposes. The statistical pattern in LCD takes a triplet of mutually dependent variables with the additional prior knowledge that one of the variables (‘*Z*’) cannot be caused by the other two (‘*X*’ and ‘*Y*’). As we will show in more detail in the Supplementary material (S1 File), any causal model that now implies a conditional independence between the variables ‘*Y*’ and ‘*Z*’ conditioned upon ‘*X*’ has a causal link from ‘*X*’ to ‘*Y*’. So, reasoning backward, if we observe such a conditional independence in our observational data, we can interpret this as evidence for a causal link from ‘*X*’ to ‘*Y*’. This causal pattern was first derived by Cooper in [29], and later independently rediscovered in the context of genome biology in [24]. This method has been applied in various papers in the biomedical research literature, such as [30, 31].

### Related models and methodologies

LCD is closely related to other, arguably more standard approaches, such as Structural Equation Modeling, mediation analysis and instrumental variable analysis. Below we explain the similarities and differences between these methods.

#### Structural equation modeling

LCD, as most methods for causal discovery, is closely related to Structural Equation Modeling (SEM). Typically, SEMs are used in a confirmatory setting, where a limited amount of specific structures are taken into consideration and compared against each other by scoring them on the available data. Causal discovery methods are used in a more exploratory setting. They assume that there is some SEM underlying the data and then aim to reason about its structure. Under particular conditions, parts of the structure can be derived from conditional (in)dependencies [20]. As explained in detail in the Appendix, LCD does exactly this for the specific case of three observed and possibly many latent variables. A key advantage of LCD over fitting different SEM structures to the data is that LCD automatically incorporates latent variables and then implicitly considers all possible models instead of just a few. This makes it possible for LCD to make generic statements about causal directions.

PLS-SEM, for partial least squares structural equation modeling, is a specific variant of structural equation modeling [32, 33]. Among others, it more explicitly handles latent variables and hence may be considered closer to the approach that we take in this paper. However, also in PLS-SEM, one starts by specifying the structure between the (latent and measured) variables, which makes it different from LCD, which aims to *infer* the (invariant parts of the) structure from observational data. Thus, by using LCD we do not have to preselect several possible models to test, as typically done with PLS-SEM. As a result, LCD can potentially infer causal statements.

#### Mediation analysis

Mediation analysis starts from the assumption that the independent variable *‘Z’* (genetic factor) causes the dependent variable *‘Y’* (impulsivity/hyperactivity) and then aims to answer the question whether the effect of *‘Z’* on *‘Y’* can be (fully) explained by the mediator *‘X’* (inattention). The important difference with the analysis underlying LCD is that LCD does not start from the assumption that there is a causal relationship, but instead aims to derive one. Nevertheless, following the analysis detailed in the Supplementary material (S1 File), it can be seen that we can only derive a causal statement if the data reveals a conditional independence, which amounts to one variable mediating the correlation between the other two.

#### Instrumental variable approaches

In so-called instrumental variable approaches [34], the genetic factor *‘Z’* is called an instrument. It can be used to estimate the causal effect of the variable *‘X’* on the variable *‘Y’* in the presence of latent confounders. A valid instrument has to satisfy various criteria, among others that its effect on the variable *‘Y’* is fully mediated by the variable *‘X’* (in more complex settings possibly controlled for other variables). The main difference with LCD is that instrumental variable analysis starts from the assumption that there is a causal effect from *‘X’* to *‘Y’* and then tries to make use of the instrument *‘Z’* to estimate or bound its strength, whereas LCD uses the instrument *‘Z’* to try and infer the existence and direction of a cause-effect relationship between *‘X’* and *‘Y’*, without attempting to estimate the causal strength of this relationship.

### Goal

The goal of this paper is to analyze whether such statistical patterns can be observed in studies of ADHD populations, and if so, what causal relationships these patterns then suggest. We will use symptom scores for inattention and hyperactivity/impulsivity as substitutes for the actual level of inattentiveness and hyperactivity/impulsivity. These then play the role of the variables ‘*X*’ and ‘*Y*’ above. For the variable ‘*Z*’ we will consider genetic variables such as gender and the *DAT1* risk haplotype. These three variables clearly satisfy the premises of LCD: they are all mutually dependent (as shown in various other studies and easily checked for the data sets analyzed in this paper) and it seems completely reasonable to assume that manipulations of inattentiveness and hyperactivity/impulsivity do not affect gender, nor the *DAT1* risk haplotype.

## Materials and Methods

### Materials

To infer causal relationships between ADHD symptoms we used three data sets, describing children, adolescents, and adults with ADHD. For each data set we only consider three variables: inattention symptom scores, hyperactivity/impulsivity symptom scores, and a genetic variable (either gender or a risk haplotype). The main rationale for choosing these data sets is availability, as explained in more detail in the discussion.

The first data set was collected for the NeuroIMAGE project [35] (see www.neuroimage.nl) and considers adolescents. We will refer to this data set as the NeuroIMAGE data set. This data set includes N = 903 participants (413 adolescents with ADHD, 228 unaffected siblings of ADHD probands, and 262 healthy control subjects) with a mean age of 16.7 years (min = 5.7 years, max = 28.6 years). The presence of ADHD symptoms was assessed by a semi-structured diagnostic interview Schedule for Affective Disorders and Schizophrenia for School-Age Children—Present and Lifetime Version (K-SADS-PL [36]) and Conners' ADHD questionnaires from multiple informants (parents and children) [37]. An algorithm was applied to create a combined symptom count from the interview and questionnaires (symptom range 0–18) (the algorithm is provided in [35]). Participants were diagnosed with ADHD if they met the full DSM-IV criteria for the disorder. For the current analyses, the sum of the symptom counts on the two symptom dimensions inattention (0–9) and hyperactivity/impulsivity (0–9) was used. In addition, we used the information on gender. In order not to complicate our analysis with ways to account for the dependencies between probands and their unaffected siblings, we ignore the siblings, leaving N = 675 subjects in total. A more detailed description of the symptom assessment and recruitment process can be found in [35].

The second data set was collected by Peking University and is publicly available as part of the ADHD-200 Sample and parts of this data were described in several papers [38–41], (http://fcon_1000.projects.nitrc.org/indi/adhd200/) and considers children. We will refer to this data set as the ADHD-200 data set. This data set includes N = 245 participants (102 children with ADHD, 143 control subjects) with a mean age of 11.7 years (min = 8.1 years, max = 17.3 years). The data set contains information about subjects’ ADHD symptom scores, disease status, gender, and IQ. Symptom scores were measured using the ADHD Rating Scale (ADHD-RS) IV [42], for which scores can range from 0 to 27 for each symptom domain. Also for this data set we will restrict our analysis to the two symptom scores and gender. We could not use the other data sets that are part of the ADHD-200 sample, because in those data sets the ADHD symptom scores were corrected for the effect of gender. More details about the ADHD-200 data sets are provided in [38].

The third data set was collected for the IMpACT project [43] and considers adults. We will refer to this data set as the IMpACT data set. This data set contains N = 164 participants (87 adults with ADHD, 77 control subjects) with a mean age of 36.6 years (min = 18.0 years, max = 63.0 years). Subjects were assessed using the Diagnostic Interview for Adult ADHD (DIVA) (www.divacenter.eu). This interview focuses on the 18 DSM-IV symptoms of ADHD and uses concrete and realistic examples to thoroughly investigate whether the symptom is present now or was in childhood. In addition, a quantitative measure of clinical symptoms was obtained using the ADHD-DSM-IV Self Rating scale [6], which has a range of scores from 0 to 9 for each symptom domain. To support the validity of the symptoms estimate based on self-reports, extra information about ADHD symptoms and impairment in childhood was obtained from parents and school reports, whenever possible. Patients were included in the study if they met the DSM-IV-TR criteria for ADHD in childhood as well as adulthood. As gender was not associated with ADHD in the adult data, we used an alternative genetic variable: the presence/absence of the *DAT1* 9/6 risk haplotype, a genetic polymorphism associated with ADHD in adulthood [43]. More detailed information about the data collection and symptom assessment can be found in the original paper by Hoogman [43]. For this type of analysis the use of DAT1 instead of gender as a genetic variable does not influence the validity of our results, since DAT1 also fulfills all the requirements of the LCD approach (DAT1 is correlated with inattention and hyperactivity/impulsivity, neither inattention nor hyperactivity can cause DAT1).

### Data analysis

The inference of causal relationships from observational data crucially depends on the detectable absence and presence of conditional dependencies between variables [21]. For random variables that follow a multivariate Gaussian distribution, conditional independence corresponds to zero partial correlation. The partial correlation between *X* and *Y* given controlling variable *Z* is defined as the correlation between the residuals *R*_*X*_ and *R*_*Y*_ resulting from the linear regression of *X* with *Z* and of *Y* with *Z*, respectively. In other words, partial correlation measures the degree of association between two random variables, with the effect of the controlling random variable removed. By measuring partial correlation it is possible to measure conditional independencies in the data.

Our symptom scores are not normally distributed and both gender and presence/absence of risk haplotype are binary variables. The standard approach of estimating conditional independencies uses Pearson partial correlation that relies on the assumption of Gaussian data. Since this assumption does not hold for our data, Pearson partial correlation is not guaranteed to represent conditional dependencies and independencies correctly for our data [44]. We therefore replaced Pearson by Spearman rank partial correlation. Technically, a standard test for zero partial correlation with Spearman correlation instead of Pearson is valid for variables that obey a so-called non-paranormal distribution [45]: a multivariate Gaussian distribution on latent variables, each of which is related to the observed variables through a monotonic transformation.

An alternative method to infer conditional independencies/dependencies from non-normally distributed data is to discretize the data at the risk of losing some statistical power and use the so-called Mantel-Haenszel test [46]. The basic idea of this test is to turn observed counts into expected counts under the assumption that there is a conditional independence and then check whether there is a significant difference between the expected and observed counts. For all three data sets we discretized the symptom scores into a binary variable using a median split, which had its threshold at 4.5. The observed counts were visualized in a cross table with a mosaic plot. A mosaic plot is an area-proportional hierarchical visualization of (typically observed) counts, composed of tiles (corresponding to the cells) created by recursive vertical and horizontal splits of a rectangle. The area of each tile is proportional to the corresponding cell entry given the dimensions of previous splits [47]. Mosaic plots are excellent tools for visualizing conditional independencies: if two variables are conditionally independent given a third, this will show in the mosaic plot through straight lines as long as the conditioning variable is not represented at the lowest level of the hierarchy.

## Results

We obtained consistent results for all three data sets. We provide a detailed description of the results for the ADHD adolescence data, including figures, in the main text. Figures for the other two data sets can be found in the Supplementary material (S2 File). A summary of the results for the three data sets is presented in Table 1.

**Table 1:** Outcome of the conditional independence tests for the three different data sets. We check both whether inattention is conditionally independent of Gender/DAT1 given hyperactivity/impulsivity (second column) and whether hyperactivity/impulsivity is conditionally independent of Gender/DAT1 given inattention (third column). R specifies the partial correlation (higher means more strongly correlated); chi-squared the Mantel—Haenszel test statistic (higher means larger deviation from independence). The p-values correspond to the null hypothesis that the two variables are conditionally independent.

Type of test	Gender/DAT1 and inattention symptom scores conditioned upon hyperactivity/impulsivity	Gender/DAT1 and hyperactivity/impulsivity symptom scores conditioned upon inattention
NeuroIMAGE		
Partial correlation test	R = 0.1235, p = 0.0013	R = -0.0008, p = 0.9826
Mantel-Haenszel test	Chi-squared = 11.37, p<0.001	Chi-squared = 0.15, p = 0.70
ADHD-200		
Partial correlation test	R = 0.18, p = 0.006	R = 0.05, p = 0.42
Mantel-Haenszel test	Chi-squared = 10.98, p = 0.001	Chi-squared = 0.47, p = 0.49
IMpACT		
Partial correlation test	R = 0.19, p = 0.02	R = -0.01, p = 0.91
Mantel-Haenszel test	Chi-squared = 11.21, p = 0.001	Chi-squared = 0.005, p = 0.95

**Fig 1:**
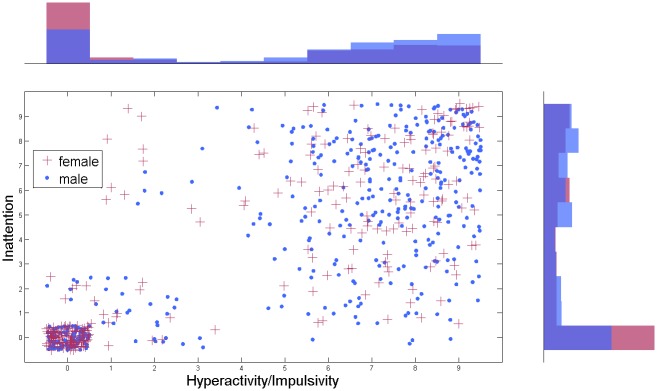
The NeuroIMAGE data set: Hyperactivity/impulsivity is plotted versus inattention symptoms for male and female. The bars indicate the histogram of the distribution. For visualization purposes random noise has been added to the discrete symptom scores.

**Fig 2:**
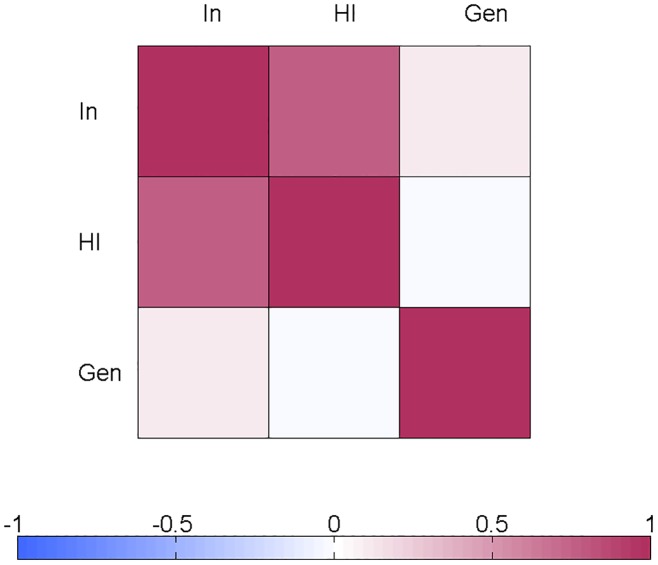
Spearman’s partial correlation coefficients for the NeuroIMAGE data set representing inattention symptoms (In), hyperactivity/impulsivity (HI) symptoms, and gender (Gen). The bar colors represent the correlation value. Every cell^(i,j)^ in the table shows Spearman partial correlation between two variables X_i_ and X_j_, conditioned on the remaining variables in the model. For example, figure shows that HI is independent of Gen given In (white square), while In depends on Gen given HI (pink square).

In Fig 1 the NeuroIMAGE data set is displayed. It can be clearly seen that all three variables are significantly correlated (see Table 2 for correlations and effect sizes). Spearman’s partial correlation between gender and hyperactivity/impulsivity symptom scores conditioned upon inattention symptom scores is negligible (Spearman R = -0.0008, p = 0.9826). However, the Spearman partial correlation between gender and inattention symptom scores conditioned upon hyperactivity/impulsivity symptom is significantly different from zero (Spearman R = 0.1235, p = 0.0013). Spearman’s rank partial correlation coefficients are visualized in Fig 2.

**Table 2:** Correlation between the three variables for three data sets and the category of the effect size (in brackets). R represents Spearman rank correlation, and p-values correspond to the null hypothesis that the two variables are independent. Effect size estimates are based on the size of the correlation observed between two variables, where small, medium, and large correlation thresholds are respectively 0.10, 0.30, and 0.50 based on Cohen’s classification [48].

	Gender/DAT1 and Inattention	Gender/DAT1 and Hyperactivity/Impulsivity	Inattention and Hyperactivity/Impulsivity
NeuroIMAGE	R = 0.187, p<0.001 (small)	R = 0.141, p<0.001(small)	R = 0.759, p<0.001 (large)
ADHD-200	R = 0.288, p<0.001 (small)	R = 0.215, p = 0.001 (small)	R = 0.679, p<0.001 (large)
IMpACT	R = 0.307, p = 0.001 (medium)	R = 0.224, p = 0.004 (small)	R = 0.764, p<0.001 (large)

**Fig 3:**
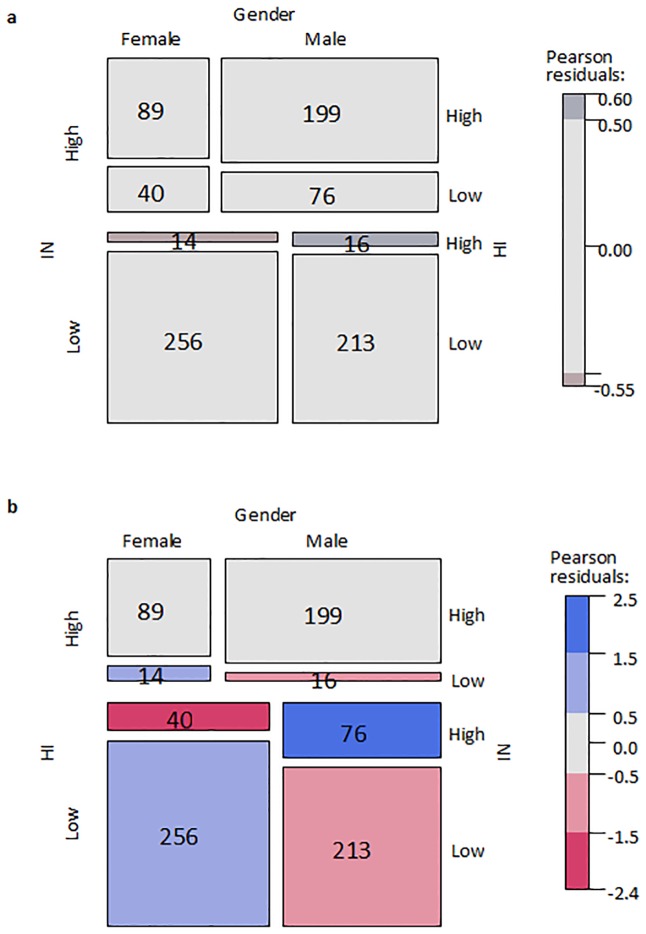
Mosaic plots of the observed counts for the NeuroIMAGE data set under the assumptions that a) hyperactivity/impulsivity symptom level and gender are conditionally independent given inattention symptom level; b) inattention symptom level and gender are conditionally independent given hyperactivity/impulsivity symptom level. The color of the cell represents the value of Pearson residuals of the Mantel-Haenszel test. Two variables are independent when the boxes proportions across categories are the same and there is a straight line that goes through these areas. For example, hyperactivity/impulsivity is independent of gender on Fig (a) when adjusted for the level of inattention, since there is no significant difference in the proportion of males and females for high and low level of hyperactivity. There is almost a straight line that divides high and low level of hyperactivity/impulsivity for both high and low level of inattention in Fig. (a). Fig. (b) shows that inattention depends on gender adjusted for the level of hyperactivity/impulsivity. There is significant difference in the proportion of females with high and low inattention when controlling for the hyperactivity/impulsivity level to the proportion of males with high and low inattention.

The Mantel-Haenszel test for discretized data provided similar results. As shown in the mosaic plots in Fig 3, there is a significant difference (chi-squared = 11.37, p<0.001) between the observed and expected scores of inattention for the different genders, conditioned upon hyperactivity/impulsivity symptom level (Fig 3a). No significant difference (chi-squared = 0.15, p = 0.70) is seen between the observed and expected scores of hyperactivity/impulsivity for different gender, conditioned upon inattention symptoms (Fig 3b). This implies that the triples in all three data sets satisfy the LCD-condition, i.e., where for a triplet of mutually dependent variables (‘*X’*, *‘Y’*, *‘Z’*) with the prior knowledge that *‘Z’* is not caused by *‘X’* and *‘Y’* we observe a conditional independency between *‘Y’* and *‘Z’* conditioned upon *‘X’*.

## Discussion

The aim of this paper was to apply a novel approach for causal discovery to improve our understanding of the strong correlation between the two symptom dimensions of ADHD. In three different and independent data sets, employing different instruments and raters to measure ADHD symptoms, and using different genetic variables, we found robust statistical evidence for a conditional independence of hyperactivity/impulsivity symptom level from a genetic variable, conditioned upon inattention symptom level. Without conditioning, the genetic variable (gender/risk haplotype) and hyperactivity/impulsivity were clearly dependent. Causal inference provides an explanation for this dependency cancellation: inattention causes hyperactivity/impulsivity.

### Interpretation

The causal statement explaining the association between hyperactivity/impulsivity and inattention asks for a careful interpretation. Obviously, inattention as well as hyperactivity/impulsivity could be caused by many factors, directly or indirectly through yet other factors. What the causal model implies is that there is a significant causal path from inattention to hyperactivity/impulsivity, but not the other way around. Furthermore, there appears to be no (unobserved) factor with a similarly relevant causal path to both inattention and hyperactivity/impulsivity, since in that case the genetic variable and hyperactivity/impulsivity should be dependent conditioned upon inattention, which contradicts with the observed conditional independence. Summarizing the above, there are factors that influence inattention directly and influence indirectly hyperactivity/impulsivity via inattention. On the other hand there are also factors that influence hyperactivity/impulsivity directly, and have no effect on inattention. The variance of the hyperactivity/impulsivity explained by the inattention ranges between 67–77% for the data sets described in this study, based on the correlation between the two variables. The rest of the variance can be explained by factors that influence hyperactivity/impulsivity directly, not via inattention.

**Fig 4:**
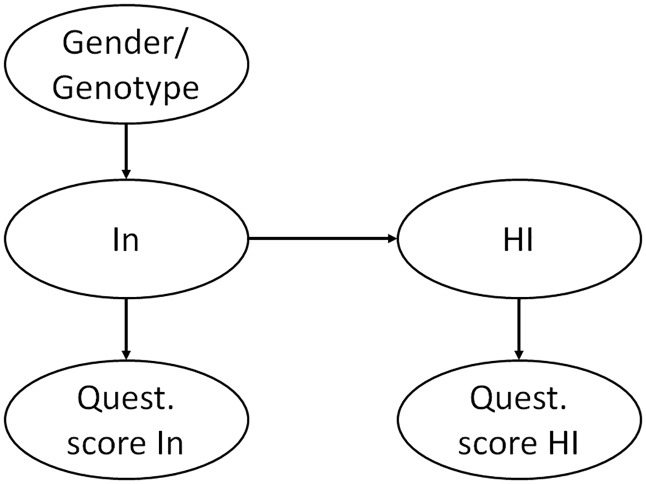
Causal relationships implied by our data for inattention (In), hyperactivity-impulsivity (HI), genetic variables (Gender or genotype), and behavioral estimates based on interview/questionnaire symptom scores.

Note also that in this causal interpretation, we treat the outcome of the interviews/questionnaires as proxy for “inattention” and “hyperactivity/impulsivity”. In fact, “inattention” and “hyperactivity/impulsivity” themselves are perhaps best viewed as hidden concepts, which can be represented as latent variables that by themselves are linked to (causing) the respective symptoms. That we find this causal link between inattention symptoms and hyperactivity/impulsivity implies that there is likely to be a latent concept (which we may call “inattention”) that is quite accurately captured by the interview/questionnaire items related to inattention and which “causes” another latent concept (which we may call “hyperactivity/impulsivity”) that is quite accurately represented by items for hyperactivity/impulsivity in the interviews/questionnaires, see Fig 4. Furthermore, when we say that one variable “causes” another, we mean that if we manage to intervene on the first variable, this will change (the probability distribution of) the second variable. A similar subtle interpretation is implicit in many practical applications of causal discovery.

### Related literature on ADHD

Early work on what we now know as ADHD in the 1940’s emphasized characteristics as hyperactivity and impulsivity as part of the so-called Minimal Brain Damage syndrome [49]. Later on, research failed to establish a firm link between hyperactivity and brain damage. Most children suffering brain damage did not develop hyperactivity, and fewer than 5% of hyperactive children appeared to suffer from brain damage [50]. During the late 60's and early 70's, the focus shifted to problems in attention regulation. Virginia Douglas and her colleagues at McGill University in Canada were among the first to demonstrate the marked attention deficits seen in these children. Douglas argued that the major deficit was the inability to “stop, look, and listen” [51]. After intense debate on what the primary features of the disorder were, the American Psychiatric Association published the DSM-III I 1980, and coined the disorder “Attention Deficit Disorder, with or without hyperactivity”. It was realized that the earlier diagnosis of hyperactivity in children does not necessarily mean that these children do not have inattention. It may well be that in small children, who have a more limited attention span than adults, inattention is harder to diagnose than hyperactivity. The results of Douglas’ research reflected the consensus that attention deficit, not hyperactivity, was the key to the disorder. The findings in our current analysis support this consensus.

The proposed model has many characteristics in common with the bi-factor model [17]. The bi-factor model allows symptoms to be associated with general factors that are common for both symptoms, and specific factors for each symptom in particular. The model proposed in this paper suggests that there are general factors that influence inattention and consequently hyperactivity, and specific factors that influence only hyperactivity. When given a causal interpretation, the bi-factor model explains a correlation between symptoms by a common cause (general factor), while our proposed model explains it by an effect from inattention to hyperactivity/impulsivity. Unfortunately, we cannot directly compare our study with the study in [17], which suggests that the bi-factor model outperforms other standard factor models of ADHD, since such analysis [17] requires symptom scores for each question, while in this study only aggregated scores per symptom were available. Furthermore, there are many slightly different variants that one could consider, each with various possible causal interpretations. In future work, we aim to extend the analysis of [17] on data with symptom scores for each question. Our current analysis strongly suggests to then explicitly incorporate gender or another genetic factor as an instrumental variable, since this may lead to larger differences between various models and could substantiate the causal relationships found through our analysis and possibly reveal others.

Our causal model is in line with findings by Willcutt and coworkers [18] in a study of ADHD heritability in adolescent twin pairs. They showed that inattention is heritable for all levels of hyperactivity/impulsivity, whereas hyperactivity/impulsivity is heritable only when the level of inattention symptoms is high. This made the authors suggest that the etiology of hyperactivity/impulsivity is different in subjects who show a high level of inattention from that in subjects with low inattention. Such a hypothesis is perfectly consistent with our causal model: there are heritable factors that cause inattention and affect hyperactivity/impulsivity downstream of that, whereas those factors that lead to high hyperactivity/impulsivity do not necessarily lead to higher inattention.

It has been found that hyperactivity/impulsivity symptoms remit more likely than inattention symptoms [52]. An obvious explanation, consistent with our model, is that those factors that directly affect hyperactivity become less prominent in adulthood, whereas the factors that affect hyperactivity through inattentions remain more or less constant. Longitudinal data are required to study such phenomena in more detail.

Considering clinical management of patients, the existence of a causal path from inattention to hyperactivity/impulsivity suggests that interventions (for example medication treatment) that decrease inattention are also likely to have a beneficial effect on the level of hyperactivity/impulsivity. On the other hand, interventions that affect hyperactivity/impulsivity cannot be expected to also have a positive effect on the level of inattention symptoms. This would further be consistent with reports that methylphenidate treatment of ADHD primarily targets attentional mechanisms by blocking the dopamine transporter in the striatum and the resulting increase in synaptic dopamine [53].

### Assumptions

As any statistical analysis, causal inference relies on several assumptions. Some of these assumptions are more fundamental, such as the assumption that we can use statistical tests to uncover the probabilistic (in)dependence relationships among the measured variables, and the assumption that reality can be properly modeled by acyclic Bayesian networks. These assumptions are discussed in detail in [29]. Note that we explicitly do not (have to) assume so-called causal sufficiency and hence do allow for the presence of latent confounders. These latent confounders could be clinical comorbidities or environmental mediators such as epigenetic mechanisms. Moreover, the fact that the observed conditional independencies were found in three independent data sets representing three different age groups and considering two different control variables, appear to rule out that these results are an artifact of a selection bias.

The selection of the appropriate data sets for the analysis was based on previous findings in our research and the availability of the data. An earlier paper [54] describes a causal analysis of data from the IMpACT study on a larger number of variables. Here we noticed, among other things, the causal link between inattention and hyperactivity/impulsivity. The analysis in this paper reveals that this causal link can also be found by restricting the analysis on the IMpACT data set to just three variables. To confirm this finding we considered the NeuroIMAGE and the ADHD-200 data sets. We did not have any other data sets available for the analysis that would satisfy the requirements mentioned in the introduction.

In this paper the ADHD case-control sample was used instead of a random sample which raises the question whether a biased sampling plan will impact the empirical associations. To answer this question we checked how the results of the conditional independence tests change if we decrease the number of ADHD cases in the sample, keeping the number of controls the same. The tests showed that if the number of ADHD cases is very small (less than 10), the correlation between the gender and symptoms becomes insignificant, due to low variation in symptoms and small sample size. Consequently, a conditional independence test between inattention and gender, conditioned on hyperactivity also becomes insignificant. When we increase the number of ADHD cases the variation in symptoms in the sample increases as well as the sample size, making the correlation between gender and symptoms more pronounced. Consequently, the dependency between inattention and gender, conditioned on hyperactivity becomes significant. However, the dependency between hyperactivity and gender, conditioned on inattention does not depend on the number of ADHD cases and is always insignificant. This analysis implies that considering a random sample instead of an ADHD case-control sample, we obtain the same sets of conditional independencies provided that the sample size is large enough. We also repeated our analysis on the siblings from the NeuroImage data set, where we found evidence for the same pattern (not reported here, because statistically less significant than the other, larger data sets).

In this paper we considered the division of ADHD into two symptom dimensions, namely inattention and hyperactivity/impulsivity. Other studies used a set of items that was much larger than the core ADHD symptoms and included items on mood, oppositional behavior and cognitive problems. These studies described a three-dimensional model splitting hyperactivity/impulsivity into separate dimensions of hyperactivity and impulsivity [55]. Future studies may extend our current work into examining causal relationships between inattention, hyperactivity and impulsivity.

## Conclusion

In this paper we discuss the robust cancellation of dependency between hyperactivity/impulsivity and a genetic factor conditioned upon inattention observed in three different data sets. It is difficult to quantify one’s confidence in a statement such as “inattention causes hyperactivity/impulsivity”, if only because it strongly depends on the typical assumptions underlying causal inference. Some of these assumptions have been debated (see e.g., the discussion in [56, 57]), and some may claim that alternative approaches are more fruitful (e.g., causal inference as a missing-data problem as proposed by [58]; see however [21]). It is clearly beyond the scope of this paper to resolve such issues. We do argue that, when one is willing to apply these methods for causal inference (see e.g., [30, 31] for similar approaches within the biomedical domain), they suggest a logical explanation for the robust cancellation of dependencies in three different studies, which follows Ockham's principle of parsimony to select the hypothesis with fewest assumptions. We further have discussed how such a causal model can be put in the historical context of the disease and may explain other findings such as those in [18] showing different etiology of the hyperactivity/impulsivity for subjects that have a high level of inattention from subjects with a low level of inattention. Last but not least, our causal model yields testable hypotheses, which may be validated in future intervention studies.

## Supporting Information

S1 File Supplementary Material. Derivation of the LCD pattern. Fig A. Set of all possible models, represented as so-called CPAGs [59] that have at least two edges. Next to each graph a set of pairwise independecies and conditional independencies is represented. X ⊥Y means that X is independent of Y; X ⊥ Y | Z means that X is independent of Y conditioned on Z.
(DOCX)

S2 File Supplementary material figures for extra data sets. Fig A. The ADHD-200 data set: Hyperactivity/impulsivity versus inattention symptoms for male and female. The bars indicate the histogram of the distribution. For visualization purposes random noise has been added to the discrete symptom scores. Fig B. Spearman’s partial correlation coefficients for the ADHD-200 data set representing inattention symptoms (In), hyperactivity/impulsivity (HI) symptoms and gender (Gen). Every cell(**i,j**) in the table shows partial correlation between two variables **X**_**i**_ and **X**_**j**_, conditioned on the remaining variables in the model. For example, figure shows that HI is independent of Gen given In (white square), while In depends on Gen given HI (pink square). Fig C. Mosaic plots of the observed counts for ADHD-200 data set under the assumption that a) inattention symptom level and gender are conditionally independent given hyperactivity/impulsivity symptom level; b) hyperactivity/impulsivity symptom level and gender are conditionally independent given inattention symptom level. The color of the cell represents the value of Pearson residuals. Fig D. The IMpACT data set: hyperactivity/impulsivity versus inattention symptoms for presence or absence of genetic risk haplotype. The bars indicate the histogram of the distribution. For visualization purposes random noise has been added to the discrete symptom scores. Fig E. Spearman’s partial correlation coefficients for the IMpACT data set representing inattention symptoms (In), hyperactivity/impulsivity (HI) symptoms and genetic risk haplotype (Gen). Every cell(**i,j**) in the table shows partial correlation between two variables **X**_**i**_ and **X**_**j**_, conditioned on the remaining variables in the model. For example, figure shows that HI is independent of Gen given In (white square), while In depends on Gen given HI (pink square). Fig F. Mosaic plots of the observed counts for IMpACT data set under the assumption that a) inattention symptom level and genetic risk haplotype DAT1 are conditionally independent given hyperactivity/impulsivity symptom level; b) hyperactivity/impulsivity symptom level and genetic risk haplotype DAT1 are conditionally independent given inattention symptom level. The color of the cell represents the value of Pearson residuals.
(DOCX)

S3 File Supplementary Material IMpACT data. (XLSX)

S4 File Supplementary Material NeuroIMAGE data. (XLSX)
