# Supplementary material for: Statistical Evidence Suggests that Inattention Drives Hyperactivity/Impulsivity in Attention Deficit-Hyperactivity Disorder
Source: PLoS One. 2016 Oct 21;11(10):e0165120. doi: 10.1371/journal.pone.0165120 (PMC5074570; doi:10.1371/journal.pone.0165120)
Supplement: S1 File — Fig A. Set of all possible models, represented as so-called CPAGs [59] that have at least two edges. Next to each graph a set of pairwise independecies and conditional independencies is represented. X ⊥Y means that X is independent of Y; X ⊥ Y | Z means that X is independent of Y conditioned on Z. (DOCX) [file pone.0165120.s001.docx]

**Supplementary material**

*Derivation of the LCD pattern*

To explain the type of reasoning and underlying assumptions in more detail, we will spell out the LCD pattern (1) for the three variables that we are interested in: inattentiveness (‘In’), hyperactivity/impulsivity (‘HI’), and a genetic factor (‘Gen’), which can be either gender or a risk haplotype such as *DAT1*. We follow essentially the same reasoning as in (1). An alternative proof can be found in (2).

Figure A displays eight models, represented as so-called complete partial ancestral graph CPAGs (3). This is an exhaustive representation of all possible models that fulfill conditions of LCD: 1. Inattention and hyperactivity/impulsivity are correlated with genetic factor; 2. Neither inattention, nor hyperactivity/impulsivity cause genetic factor. We only consider models that have at least two edges, since with less than two edges at least one of the variables will be independent of the other two, clearly violating the fact that all three variables are mutually dependent. Each CPAG, short for ‘complete partial ancestral graph’, by itself represents a whole class of possible causal models, not only over the observed variables but also over unknown latent variables. In these graphs, “*X*→*Y*” means that there must be a causal path from *X* to *Y* in the underlying causal model, “*X*↔*Y*” that there is no causal path from X to Y or from Y to X, so there must be a latent common cause affecting both *X* and *Y*. Circle marks are wild cards, that is, “*X*∘→*Y*” means either “*X*→*Y*” or “*X*↔*Y*”, and “*X*∘−∘*Y*” any of “*X*→*Y*”, “*X*←*Y*”, and “*X*↔*Y*” (note that here, in technical terms, we do allow for the possibility of latent variables, i.e., do not assume so-called causal sufficiency, but do assume that there is no selection bias and there cannot be any cycles; we will get back to these assumptions in the discussion). In our case, the assumption that no other observed variable in the model (neither inattention, nor hyperactivity/impulsivity) causes ‘Gen’ implies that when there is an edge between ‘Gen’ and, for example, ‘In’, it always comes with an arrowhead at ‘In’ and typically a circle mark at ‘Gen’. This implements our assumption that ‘In’ cannot cause ‘Gen’, without excluding the possibility that there is a latent common cause affecting the two (a circle mark at ‘Gen’). For example there can be a latent gene that causes both ‘In’ and ‘Gen’.

Reasoning forward, each CPAG now implies a set of (conditional) dependencies and independencies. They can be derived using a general property called *m*-separation (4), here with just three variables arguably also through common sense. The implied (conditional) dependencies for each possible combination of variables are shown in the columns on the right, next to each of the CPAGs. Here, for example, “In ⊥ HI | Gen” means that ‘In’ is independent of ‘HI’ when conditioned upon ‘Gen’. Graph (a) implies no (conditional) independencies at all. Graphs (b1) through (d2) are potentially more interesting: they all at least suggest one (conditional) independence. Letters refer to the models with the same set of edges, while numbers distinguish different edge directions in Figure A. For example, although model (b1) and (b2) represents two similar models with the same skeleton (an edge between ‘Gen’ and ‘HI’ and ‘Gen’ and ‘In’), and the same set of conditional independencies, they declare possibly different edge directions. Three causal models are possible a) causal effect from ‘Gen’ to ‘In’ and ‘HI’ (possible for both Figure b1 and b2); b) causal effect from ‘Gen’ to ‘In’ and a common cause between ‘Gen’ and ‘HI’ (Figure b1); c) causal effect from ‘Gen’ to ‘HI’ and a common cause between ‘Gen’ and ‘In’ (Figure b2).

Now, if we observe a particular pattern of dependencies and independencies in the data, we can reason backward to tell which graph(s) can explain these. If indeed all variables are mutually dependent, graphs (b3), (c2), and (d2) drop out because they imply marginal independence between ‘In’ and ‘HI’. If all variables are mutually dependent, but we still have a conditional independence, graph (a) drops out, and only one of (b1), (b2), (c1), or (d1) applies, depending on which conditional independence holds true. If we find that ‘In’ and ‘HI’ are conditionally independent given ‘Gen’, we can conclude that ‘Gen’ must cause either ‘In’ or ‘HI’, but cannot tell which one. However, when one of the other two conditional independencies holds true, we have either (c1) or (d1), and in both cases we can infer a causal statement.

If according to the data ‘HI’ is independent of ‘Gen’ conditioned upon ‘In’ (model c1 in Figure A), LCD concludes that there must be a causal path from ‘In’ to ‘HI’ in any underlying causal model that can explain this particular pattern of (conditional) dependencies and independencies. This is exactly the pattern of conditional independencies/dependencies that was found in the three data sets discussed in the paper.

| CPAG | In ⊥ HI | Gen ⊥ HI | Gen ⊥ In | In ⊥ HI \| Gen | Gen ⊥ HI \| In | Gen ⊥ In \| HI |
| --- | --- | --- | --- | --- | --- | --- |
| (a)  Gen  HI  In | No | No | No | No | No | No |
| (b1)  HI  In  Gen | No | No | No | Yes | No | No |
| (b2)  HI  In  Gen | No | No | No | Yes | No | No |
| (b3)  HI  In  Gen | Yes | No | No | No | No | No |
| (c1)  In  HI  Gen | No | No | No | No | Yes | No |
| (c2)  In  HI  Gen | Yes | No | No | No | Yes | No |
| (d1)  In  HI  Gen | No | No | No | No | No | Yes |
| (d2)  In  HI  Gen | Yes | No | No | No | No | Yes |

Figure A. Set of all possible models, represented as so-called CPAGs (3) that have at least two edges. Next to each graph a set of pairwise independecies and conditional independencies is represented. X ⊥Y means that X is independent of Y; X ⊥ Y | Z means that X is independent of Y conditioned on Z.

**Bibliography**

1. Cooper GF. A simple constraint-based algorithm for efficiently mining observational databases for causal relationships. 1997;1:203-24.

2. Chen LS, Emmert-Streib F, Storey JD. Harnessing naturally randomized transcription to infer regulatory relationships among genes. Genome Biol. 2007;8(10).

3. Zhang J. On the completeness of orientation rules for causal discovery in the presence of latent confounders and selection bias. Artificial Intelligence. 2008;172(16-17).

4. Richardson T, Spirtes P. Ancestral graph Markov models. Annals of Statistics. 2002:962-1030.

5. Angrist JD, Imbens GW, Rubin DB. Identification of causal effects using instrumental variables. Journal of the American statistical Association. 1996;91(434):444-55.
