# Supplementary material for: Statistical Evidence Suggests that Inattention Drives Hyperactivity/Impulsivity in Attention Deficit-Hyperactivity Disorder
Source: PLoS One. 2016 Oct 21;11(10):e0165120. doi: 10.1371/journal.pone.0165120 (PMC5074570; doi:10.1371/journal.pone.0165120)
Supplement: S2 File — Fig A. The ADHD-200 data set: Hyperactivity/impulsivity versus inattention symptoms for male and female. The bars indicate the histogram of the distribution. For visualization purposes random noise has been added to the discrete symptom scores. Fig B. Spearman’s partial correlation coefficients for the ADHD-200 data set representing inattention symptoms (In), hyperactivity/impulsivity (HI) symptoms and gender (Gen). Every cell(i,j) in the table shows partial correlation between two variables Xi and Xj, conditioned on the remaining variables in the model. For example, figure shows that HI is independent of Gen given In (white square), while In depends on Gen given HI (pink square). Fig C. Mosaic plots of the observed counts for ADHD-200 data set under the assumption that a) inattention symptom level and gender are conditionally independent given hyperactivity/impulsivity symptom level; b) hyperactivity/impulsivity symptom level and gender are conditionally independent given inattention symptom level. The color of the cell represents the value of Pearson residuals. Fig D. The IMpACT data set: hyperactivity/impulsivity versus inattention symptoms for presence or absence of genetic risk haplotype. The bars indicate the histogram of the distribution. For visualization purposes random noise has been added to the discrete symptom scores. Fig E. Spearman’s partial correlation coefficients for the IMpACT data set representing inattention symptoms (In), hyperactivity/impulsivity (HI) symptoms and genetic risk haplotype (Gen). Every cell(i,j) in the table shows partial correlation between two variables Xi and Xj, conditioned on the remaining variables in the model. For example, figure shows that HI is independent of Gen given In (white square), while In depends on Gen given HI (pink square). Fig F. Mosaic plots of the observed counts for IMpACT data set under the assumption that a) inattention symptom level and genetic risk haplotype DAT1 are conditionally independent given hy [file pone.0165120.s002.docx]

**Supplement materials**


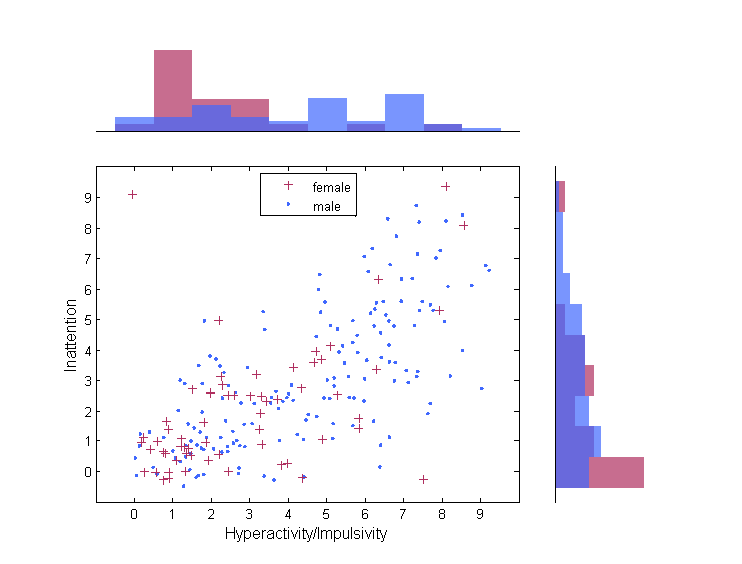


Figure A. The ADHD-200 data set: Hyperactivity/impulsivity versus inattention symptoms for male and female. The bars indicate the histogram of the distribution. For visualization purposes random noise has been added to the discrete symptom scores.


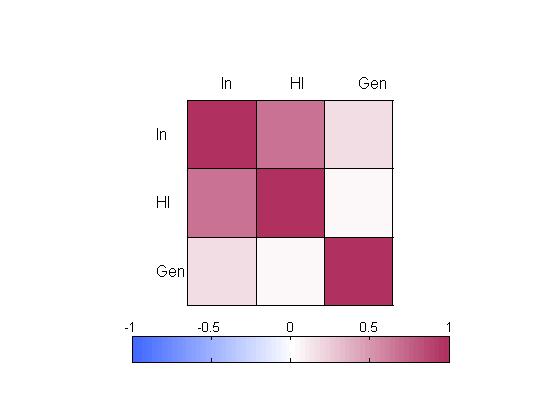


Figure B. Spearman’s partial correlation coefficients for the ADHD-200 data set representing inattention symptoms (In), hyperactivity/impulsivity (HI) symptoms and gender (Gen). Every cell$(i,j)$ in the table shows partial correlation between two variables $X_{i}$ and $X_{j}$, conditioned on the remaining variables in the model. For example, figure shows that HI is independent of Gen given In (white square), while In depends on Gen given HI (pink square).


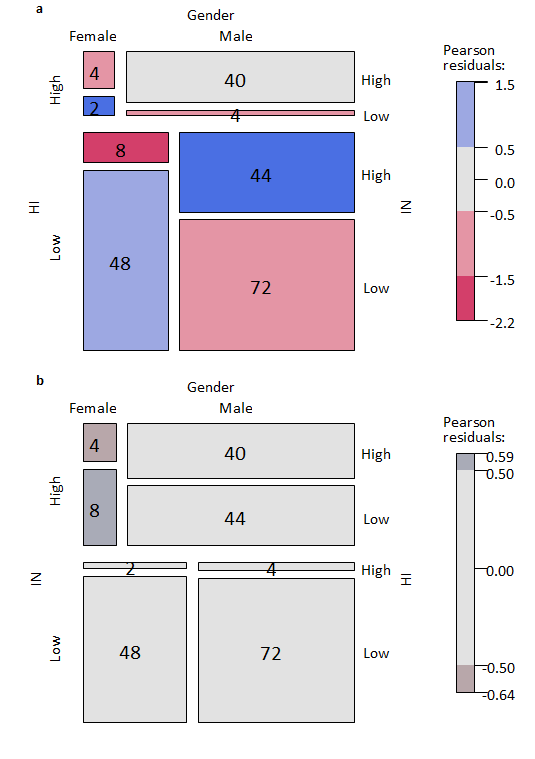


Figure C. Mosaic plots of the observed counts for ADHD-200 data set under the assumption that a) inattention symptom level and gender are conditionally independent given hyperactivity/impulsivity symptom level; b) hyperactivity/impulsivity symptom level and gender are conditionally independent given inattention symptom level. The color of the cell represents the value of Pearson residuals.


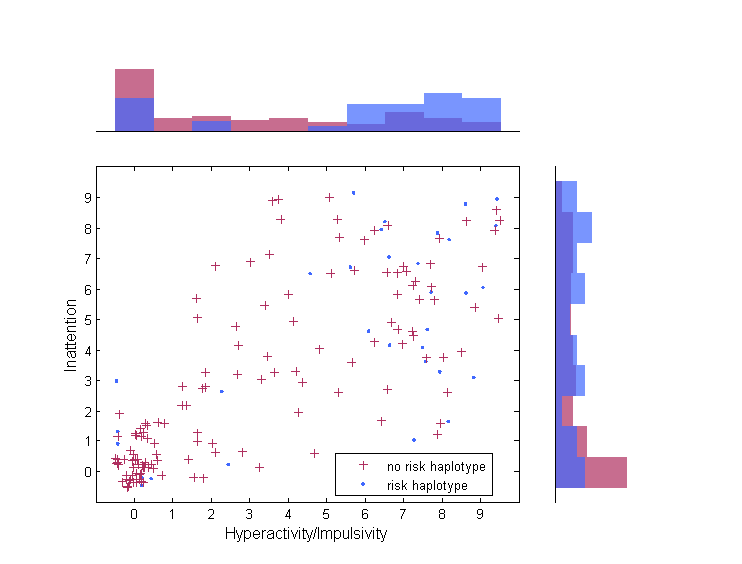


Figure D. The IMpACT data set: hyperactivity/impulsivity versus inattention symptoms for presence or absence of genetic risk haplotype. The bars indicate the histogram of the distribution. For visualization purposes random noise has been added to the discrete symptom scores.


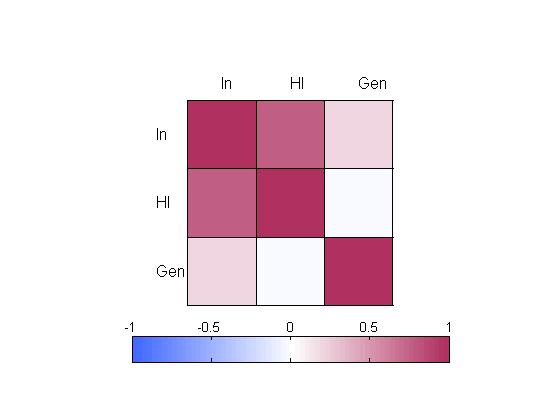


Figure E. Spearman’s partial correlation coefficients for the IMpACT data set representing inattention symptoms (In), hyperactivity/impulsivity (HI) symptoms and genetic risk haplotype (Gen). Every cell$(i,j)$ in the table shows partial correlation between two variables $X_{i}$ and $X_{j}$, conditioned on the remaining variables in the model. For example, figure shows that HI is independent of Gen given In (white square), while In depends on Gen given HI (pink square).


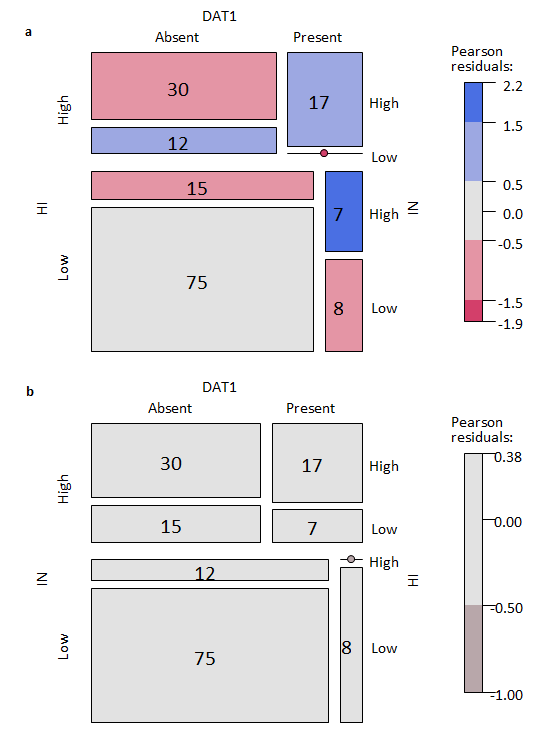


Figure F. Mosaic plots of the observed counts for IMpACT data set under the assumption that a) inattention symptom level and genetic risk haplotype DAT1 are conditionally independent given hyperactivity/impulsivity symptom level; b) hyperactivity/impulsivity symptom level and genetic risk haplotype DAT1 are conditionally independent given inattention symptom level. The color of the cell represents the value of Pearson residuals.
